# Formulation Development of a pH-Adjusted, Preservative-Free 1% 5-Fluorouracil Eye Drop Compounded from Injectable Concentrate: A Risk-Assessed Magistral Formulation and 28-Day Stability Case Study

**DOI:** 10.3390/ph19101600

**Published:** 2026-10-09

**Authors:** Péter Vajda, Gábor Bartus, Máté Csapó, István László Horváth, Márton Vajna, Szilvia Sebők, Romána Zelkó

**Affiliations:** University Pharmacy Department of Pharmacy Administration, Faculty of Pharmacy, Semmelweis University, Hőgyes Endre u. 7-9, 1092 Budapest, Hungary; vajda.peter@semmelweis.hu (P.V.); bartus.gabor@semmelweis.hu (G.B.); csapo.mate.attila@semmelweis.hu (M.C.); horvath.istvan@semmelweis.hu (I.L.H.); vajna.marton@semmelweis.hu (M.V.)

**Keywords:** 5-fluorouracil, ocular surface squamous neoplasia, magistral preparation, pharmaceutical compounding, risk assessment, topical chemotherapy, pharmacy practice, ophthalmic solution, stability, hospital pharmacy, quality target product profile

## Abstract

**Background:** No licensed ophthalmic 5-fluorouracil (5-FU) product is available in Hungary, and published compounded 5-FU eye drops are alkaline dilutions of the injectable concentrate. We report a formulation development and 28-day storage-stability case study of a pH-adjusted, preservative-free 1% 5-FU eye drop prepared under Council of Europe Resolution CM/Res(2016)1. **Methods:** Five injectable concentrates were characterised for pH, and one was titrated with 0.1 N hydrochloric acid in a preservative-free ophthalmic vehicle to pH 7.30–7.60. Three 10 mL batches were prepared aseptically and release-tested; drop volume and delivered dose were determined on one batch, which was also followed for 28 days at 15–25 °C and 2–8 °C by inspection, pH, sterility and comparative HPLC-UV assay (single determinations). **Results:** All concentrates were alkaline (pH 8.95–9.18). The selected formulation reached pH 7.43; individual doses (mean 0.435 mg per drop) were within ±8% of the mean. At 15–25 °C, samples remained clear and sterile, with pH 7.41–7.51 and assay ≥ 97% of initial, without directional trend. At 2–8 °C, precipitate formed from day 7 and the supernatant assay fell to 89.1% by day 28. **Conclusions:** The preparation remained within specification for 28 days at controlled room temperature but not under refrigeration, consistent with, though not proven to result from, the lower solubility of un-ionised 5-FU. The 28-day period is provisional, not a validated shelf-life; in-use stability, osmolality and viscosity were not established.

## 1. Introduction

Ocular surface squamous neoplasia (OSSN; conjunctival/corneal malignancy, ICD-10 C69.0) threatens ocular integrity and vision. Surgical excision has traditionally been first-line therapy, but topical 5-fluorouracil (5-FU) eye drops have emerged as a less invasive, organ-preserving alternative for primary treatment and post-surgical prophylaxis [1,2,3].

Topical 5-FU, a cytostatic antimetabolite acting mainly on epithelial and sub-epithelial cells, achieves complete regression in 70–90% of cases with 0.5% and 1% formulations, although roughly one-third of patients experience recurrence [1,2,3,4,5]. It is commonly given as a pulsed regimen of one drop four times daily for 4 days, repeated after an interval of 3–4 weeks [4,6], and its local adverse effects (burning, tearing, periorbital erythema and occasional punctal stenosis) are usually manageable [1,3,4,7,8].

In summer 2025, clinicians at our tertiary university hospital requested a 1% (10 mg/mL) 5-FU eye drop for a patient with conjunctival malignancy who lacked access to a commercial ophthalmic 5-FU product. No licensed 5-FU eye drop or harmonised EU formula exists; only injectable (Fluorouracil-TEVA, 5-Fluorouracil Sandoz), topical dermatological solution (Verrumal) and cream (Efudix) products are marketed, none intended for ophthalmic use [9,10,11,12]. This necessitated development of an evidence-based magistral preparation compliant with Council of Europe quality and safety standards, CM/Res(2016)1 [13,14,15].

Published pharmaceutical data on compounded 5-FU eye drops remain limited, and every preparation reported to date is a simple dilution of the injectable concentrate in an isotonic aqueous vehicle. Fuhrman et al. first described the stability of an extemporaneously compounded 5-FU ophthalmic solution [16]. Komm et al. subsequently characterised compounded 5-FU at 20–50 mg/mL in 0.9% sodium chloride and 5% dextrose using a stability-indicating HPLC method, observing precipitation in certain containers between days 10 and 15 [17]. Most recently, Antonietti et al. showed that a 1% (10 mg/mL) eye drop prepared by diluting the injectable concentrate in preservative-free 0.9% sodium chloride retained 97.5–103.6% of its initial potency for 12 months, at a pH that remained between 8.55 and 8.82 throughout [18]. None of these preparations was adjusted towards the physiological tear-film pH, none used a dedicated ophthalmic vehicle other than saline, and none was developed within a formal, documented pharmaceutical risk-assessment framework.

The present work therefore makes no claim of priority for stability data on compounded 5-FU eye drops. It is presented as a formulation development and 28-day stability case study. Its contribution lies in the documented, risk-assessed design of a pH-adjusted magistral preparation under CM/Res(2016)1, framed by a quality target product profile; in the comparison of injectable 5-FU concentrates from five manufacturers as starting materials, which to our knowledge has not previously been reported; and in the identification of the acid–base speciation of 5-FU as the property that couples ocular tolerability to physical stability. We present the pharmaceutical design, risk assessment, compounding, quality control and real-time stability characterisation of a standardised 1% 5-FU eye drop. Formal Quality-by-Design elements such as a design space were not developed, for the reasons given in Section 2.1.

## 2. Results

### 2.1. Risk Assessment Summary

The qualitative structured assessment (Table 1), performed as an alternative method permitted under CM/Res(2016)1 rather than by the model procedure of Note 1 of the Resolution (Section 4.1), confirmed an overall high-risk classification, driven by the hazard profile of 5-FU, the zero-tolerance ophthalmic route and aseptic-process complexity, partially offset by controlled compounding infrastructure, pharmaceutical-grade materials, small batch volumes and trained personnel. Compounding was restricted to the institutional cytostatic laboratory; home compounding was deemed unsafe, and unit-dose containers, while microbiologically preferable, were judged economically impractical at current volumes. Inadvertent refrigeration by the patient, identified from the stability results, was added to the assessment as a use-related risk (Table 1).

Because the work is positioned as a formulation development case study rather than as a full Quality-by-Design programme, those elements of ICH Q8(R2) [20] that are proportionate to a magistral preparation were applied. A quality target product profile (QTPP) was defined, and each quality attribute was classified as critical or non-critical according to its potential impact on patient safety and efficacy. The profile, the justification of criticality and the extent to which each attribute was characterised in the present study are given in Table 2.

The principal critical process parameter (CPP) is the volume of 0.1 N hydrochloric acid added per 2 mL of concentrate, expressed in this work as the vehicle-to-acid ratio at a fixed total volume of 10 mL. It determines the final pH directly and, through pH, the proportion of un-ionised 5-FU and hence the margin against precipitation (Section 2.3); through the sodium chloride generated in situ it also contributes to osmolality. Secondary process parameters are the order of addition (vehicle before acid, with mixing, to limit local pH excursions during titration), the compounding and storage temperature (physical stability) and the aseptic-processing parameters (sterility). The CPP was controlled by a fixed composition, in-process pH measurement with a stepwise correction protocol (Section 4.6) and a release specification of pH 7.30–7.60.

No design space was constructed. Only two vehicle-to-acid ratios were evaluated in the optimisation series, which is insufficient to model the CPP–CQA relationship, and a design space would be disproportionate for a preparation made in 10 mL batches for fewer than 20 units per year. For a magistral preparation of fixed composition, the control strategy rests instead on the fixed formula, in-process pH control and end-product release testing, consistent with the risk-based approach of CM/Res(2016)1 [13,15].

### 2.2. Inter-Manufacturer Variability and pH Optimisation

The pH of the injectable concentrates before and after addition of a fixed 3 mL aliquot of 0.1 N hydrochloric acid is given in Table 3.

All concentrates were characteristically alkaline (pH 8.95–9.18), reflecting formulation for storage stability of the active substance [22]. The spread across four manufacturers was 0.23 pH units (mean 9.05, SD 0.10). Because each value is a single reading on one lot per manufacturer, this spread cannot be separated from lot-to-lot and measurement variability, neither of which was determined, and no inferential comparison between manufacturers was possible; we therefore claim neither that the sources differ nor that they are equivalent. The practically relevant finding does not depend on this distinction: every concentrate examined lay 1.35–1.58 pH units above the upper limit of the tear-film range, a difference far too large to be attributed to measurement variation and consistent with the alkaline sodium-salt formulation common to these products [22]. Because only the Accord concentrate was titrated to the target, the titration behaviour of the other concentrates close to pH 7.3–7.6 was not established; similar initial pH values do not guarantee similar buffering near the target. The formula is therefore defined by its target pH rather than by a fixed acid volume (Section 2.7). A fixed 3 mL acid aliquot reduced pH by approximately 1.0 unit regardless of source, but the resulting pH (8.03–8.16) remained above the physiological tear-film range of 7.3–7.6 [21,23], so that a larger and source-independent acid volume was required.

Two optimised formulations were therefore prepared with larger acid volumes at the expense of vehicle volume, and their measured pH values are compared in Table 4.

Formulation E was selected as the final composition on the basis of its pH: the choice was a target-pH selection between two candidates rather than a comprehensive optimisation, and Formulation F was not carried into stability testing. Of the two optimised formulations, only Formulation E (pH 7.43) lies within the physiological tear-film range of 7.3–7.6 [21,23] and within the reported pH optimum for 5-FU solution stability of 7.4–8.0 [22]; Formulation F (pH 7.19) lies below both, and sits closer to the region below pH 6–7 in which degradation of 5-FU accelerates [22]. No formal estimate of pH measurement uncertainty was made (Section 4.6); the rejection of Formulation F, 0.11 units below the lower specification limit, therefore rests on its measured value rather than on a statistical comparison. The release specification for pH was accordingly set at 7.30–7.60. This range reproduces the physiological tear-film pH range [21,23]; its lower limit keeps the preparation well above the region of accelerated degradation, and it overlaps the lower part of the reported stability optimum of 7.4–8.0 [22]. All pH values recorded for Formulation E during room-temperature storage (7.41–7.51; Table 5) lay within this range. A range extending towards pH 8 would widen the solubility margin (Section 2.3) at the cost of a larger departure from tear-film pH; it was not evaluated. Over the acid volumes examined (3, 4 and 5 mL, the first with different concentrate sources), pH decreased monotonically with added acid, which permitted fine adjustment; with so few points, no quantitative model of this relationship is claimed; only two vehicle-to-acid ratios were evaluated, and alternative buffer systems were not explored (Section 3.9 and Section 3.10).

### 2.3. Formulation Rationale and Acid–Base Speciation

The 1% (10 mg/mL) concentration reflects the effective 0.5–1% range reported in the clinical literature [1,2,3] and clinician and protocol precedent, balancing efficacy against local irritation. The preservative-free vehicle avoids the sensitisation and ocular-surface toxicity associated with preservatives. The 0.1 N hydrochloric acid titrant enables precise pH control, avoiding over-acidification, which accelerates degradation below pH 6, while approaching the reported 5-FU stability optimum of pH 7.4–8.0 [22].

The mechanistic basis of this pH adjustment merits explicit statement, because it bears directly on the physical stability reported in Section 2.5. 5-Fluorouracil is a weak acid with a first pKa of approximately 8.0, and the reported aqueous solubility of the un-ionised form is approximately 12.2 g/L (12.2 mg/mL) at 20 °C [24]. Injectable concentrates are formulated at pH 8.5–9.5 precisely because at that pH the drug is present predominantly as the freely soluble sodium salt. Titration of a 10 mL batch with 4 mL of 0.1 N hydrochloric acid delivers approximately 0.4 mmol of protons against approximately 0.77 mmol of 5-FU, so that lowering the pH from about 9.0 to about 7.4 converts a substantial proportion of the ionised drug to the un-ionised free acid. Because the acid–base contribution of the vehicle and the exact base content of the concentrate are not known, the speciation is calculated below from the measured pH rather than from this stoichiometry.

The speciation can be estimated explicitly. By the Henderson–Hasselbalch relationship, the un-ionised fraction of a monoprotic weak acid is f = 1/(1 + 10^(pH−pKa)^). At the measured pH of 7.43 and a thermodynamic pKa of 8.0 [24], f = 0.79: about 79% of the drug (7.9 mg/mL) is un-ionised and about 21% is present as the anion. Two assumptions should be stated. First, the calculation uses a thermodynamic pKa; at the ionic strength of the finished preparation, estimated at 0.07–0.13 mol/L from the sodium chloride generated in situ, the residual sodium 5-fluorouracilate and the vehicle, the Davies approximation lowers the apparent pKa by about 0.1 unit, giving f ≈ 0.75 (7.4–7.5 mg/mL). Second, it treats the measured pH as exact; over the range of pH values recorded for Formulation E during room-temperature storage (7.41–7.51; Table 5), f remains between 0.76 and 0.80. On these assumptions, un-ionised 5-FU is present at 7.4–7.9 mg/mL at pH 7.43, approximately 60–65% of its reported aqueous solubility of 12.2 mg/mL at 20 °C [24]. Reported values of the first pKa of 5-FU, however, range from about 7.5 to 8.0 [24,25]. With a pKa of 7.5, f = 1/(1 + 10^(7.43−7.5)^) = 0.54 (5.4 mg/mL, about 44% of the reported solubility). Across the literature range, un-ionised 5-FU therefore accounts for roughly 54–79% of the drug, or about 44–65% of its solubility at 20 °C; the conclusion that the preparation is well below saturation at room temperature does not depend on which literature pKa is adopted.

The un-ionised fraction alone therefore does not place the preparation at saturation at room temperature. Several factors, none of which was quantified here, would narrow the margin further: (i) temperature—the aqueous solubility of 5-FU falls on cooling, and for weak acids of this type the pKa rises on cooling, increasing the un-ionised fraction, so that both effects act towards supersaturation at 2–8 °C; (ii) ionic strength—the sodium chloride generated in situ (about 40 mmol/L) may reduce the solubility of the neutral species by salting-out; (iii) excipients—polymeric components of the vehicle may either raise the apparent solubility or promote nucleation; and (iv) process—local over-acidification during titrant addition may transiently exceed saturation and generate nuclei that grow on cooling. A margin of at least one-third of the saturation concentration at room temperature is consistent with the absence of precipitation at 15–25 °C over 28 days, and a reduced or eliminated margin at 2–8 °C is consistent with the precipitation observed on refrigeration (Section 2.5). This is a post hoc interpretation, constructed after the refrigerated behaviour had been observed, and it is presented as a hypothesis rather than as an independent prediction.

The same composition permits a calculated, but not measured, estimate of osmolarity. Assuming that the sodium content of the concentrate is approximately equimolar with 5-FU (0.70–0.77 mmol per 2 mL) and ideal behaviour, the concentrate and titrant contribute about 190 mOsm/L (5-FU species about 77, sodium 70–77 and chloride 40 mOsm/L), to which the vehicle adds 0.4 times its own osmolality. The vehicle itself (Section 4.2) contains only the phosphate buffer as osmotically active solute, about 150 mOsm/L (disodium phosphate about 126 and sodium dihydrogen phosphate about 26 mOsm/L), and therefore contributes about 60 mOsm/L. The finished preparation is thus estimated at about 250 mOsm/L ideal, or about 230–240 mOsm/kg after allowance for non-ideality, i.e., mildly hypotonic relative to the tear-film range of 290–300 mOsm/kg [21]. The estimate depends on the assumed sodium content and on the vehicle composition and is not a substitute for measurement; no claim of isotonicity is therefore made (Section 3.9).

### 2.4. Drop Volume and Delivered Dose

Gravimetric determination (45 drops, total mass 1.962 g; replicate structure in Section 4.7) gave a mean drop mass of 0.0436 g and a mean drop volume of 43.5 µL (density 1.002 g/mL; RSD 4.1%). At 10 mg/mL this corresponds to a nominal dose of 0.435 mg 5-FU per drop. In the separate dose-uniformity series of 20 drops (Section 4.7), the delivered dose was 0.435 ± 0.022 mg per drop (range 0.40–0.47 mg; RSD 5.1%). Evaluated by analogy with the acceptance criteria of Ph. Eur. 2.9.27 for delivered doses from multidose containers (not more than 2 of 20 individual doses deviating from the mean by more than 10%, and none by more than 20%) [26], the series complied: no individual dose deviated from the mean by more than 8%. The experiment characterises the reproducibility of the delivered drop volume and of the calculated nominal dose per drop; it is not a content-uniformity test, because 5-FU was not assayed in individual drops.

The two relative standard deviations refer to different measurements and are reported separately: 4.1% describes the dispersion of drop volume within the 45-drop gravimetric series, whereas 5.1% describes the dispersion of delivered dose across the separate series of 20 drops used for dose uniformity. Since delivered dose is the product of a constant concentration and a variable volume, the two figures would be expected to converge on repeated determination; both indicate good reproducibility of the dropper. Within the dose-uniformity series, the lowest and highest individual doses (0.40 and 0.47 mg) lie 1.6 SD below and above the mean, i.e., within ±8% of it, so that no individual value qualifies as an outlier by a Grubbs or ±3 SD criterion, and every individual dose lies within the ±10% band around the mean.

A drop volume of 43.5 µL substantially exceeds the 7–10 µL capacity of the human conjunctival sac, so that the greater part of each instilled drop is cleared through the nasolacrimal duct or lost as periocular spillage. For a cytotoxic instillate, this is not solely a matter of economy, and the clinical implications are considered in Section 3.4.

### 2.5. Stability

The appearance, pH and assay of Formulation E over 28 days under both storage conditions are summarised in Table 5. The values are single determinations on one batch (Section 4.3).

At 15–25 °C, the formulation remained clear, colourless to slightly yellow and free of visible particles throughout 28 days, with pH stable between 7.41 and 7.51—within the 7.30–7.60 release specification at every timepoint—and assay at or above 97.0% of the initial value. At 2–8 °C, opalescence appeared from day 4 and precipitate from day 7, and the assay of the supernatant declined progressively to 89.1% by day 28. Refrigeration is therefore contraindicated for this preparation (freezing was not studied and is excluded as a precaution only): a preparation containing precipitate cannot deliver a uniform dose, irrespective of the fate of the drug that has left solution. Because patients may nonetheless refrigerate the product, the handling protocol requires inspection before each use and discard of any container showing cloudiness or particles, without attempting to redissolve the precipitate by warming (Section 4.12). This instruction is deliberately independent of whether the precipitation proves reversible, since redissolution cannot be verified by the patient.

The decline in the refrigerated arm coincides in time with the appearance of opalescence and then precipitate, and is consistent with the drug leaving solution rather than undergoing chemical decomposition, as would follow from the solubility argument of Section 2.3. Because redissolution on re-warming was not tested and the assay was not validated as stability-indicating, the two explanations cannot be separated on the present data, and the observation is reported here as an apparent loss of assay accompanying visible precipitation rather than as measured degradation.

Three observations from the existing data bear on this distinction without resolving it. First, the refrigerated assay values fell at every successive timepoint, from 100.0% to 89.1% of the initial value (exploratory least-squares slope −0.35% per day), whereas the room-temperature values at the same pH fluctuated between 97.0% and 100.0% without directional trend (exploratory slope −0.07% per day); since chemical degradation would be expected to be slower, not faster, at 2–8 °C than at 15–25 °C, a decline confined to the colder arm is difficult to attribute to hydrolytic degradation alone. Second, if the loss is entirely physical, the 10.9% shortfall at day 28 corresponds to approximately 1.1 mg/mL, or about 11 mg per 10 mL container, held in the precipitate; this is an upper-bound mass balance by difference, not a measurement. Third, the pH of the refrigerated arm drifted slightly upwards (7.45 to 7.53), the direction expected when the un-ionised acid leaves solution; however, the refrigerated values (7.44–7.53) largely overlap those recorded at room temperature (7.41–7.51), so that, with single determinations, the drift cannot be distinguished from the variability observed in the room-temperature arm (Section 4.6). Because the precipitate was not analysed and redissolution was not tested, the mechanism is stated as a hypothesis, and the handling protocol has been written so that it does not depend on the answer. Two controls that would test the attribution directly—re-warming and re-assay of precipitated samples, and an unadjusted (pH ≈ 8–9) comparator in the same vehicle and container—were not included, so the attribution to the un-ionised species remains an inference. Indirect support comes from the report that an unadjusted 1% preparation in saline (pH 8.55–8.82) showed no precipitation during 6 months at 2–8 °C [18], although that preparation differed in vehicle and container.

In the room-temperature arm, the assay values (100.0, 98.0, 100.0, 97.0, 99.0, 98.0 and 97.0% of the initial value) are non-monotonic and show no directional trend over 28 days (mean 98.4%, SD 1.3%; exploratory least-squares slope −0.07% per day); the variation is of the order commonly associated with HPLC assay variability, although with single determinations analytical variation cannot be separated from genuine change. Kinetic modelling of these data was therefore not attempted, and no storage period beyond the 28 days actually tested is extrapolated. Because each timepoint is represented by a single determination on one batch, the regression slopes quoted in this section are exploratory descriptors of direction and magnitude only; no hypothesis tests or confidence limits are reported, and no statistical shelf-life estimation of the ICH Q1E type [27] was attempted (Section 4.8). The 28-day storage period assigned to the unopened preparation is therefore a provisional, institution-specific assignment that rests on the direct observation of compliant appearance, pH and assay at every timepoint up to day 28, together with the microbiological results of Section 2.6. It is not a formally validated shelf-life, which would require replicate determinations on several batches and a validated, stability-indicating assay (Section 3.5 and Section 3.10).

### 2.6. Microbiological Results

The preparation remained microbiologically stable for the full duration of the study. Sterility was confirmed at every timepoint examined over the 28-day period under both storage conditions, in accordance with Ph. Eur. 2.6.1 [28]. Bacterial endotoxin at the time of compounding was below 0.05 EU/mL, well below the internal limit of 0.2 EU/mL. No visible particles were detected in the room-temperature arm at any timepoint. These results characterise the preparation as stored; in-use microbiological performance was not studied (Section 3.6).

### 2.7. Institutional Magistral Formula

The resulting institutional formula, as prepared, released and labelled, is set out in Table 6. The formula is defined as titration to pH 7.30–7.60: the acid volume of 4 mL is the nominal volume established for the Accord concentrate used in this study, and the vehicle volume is adjusted so that the total remains 10 mL; with any other concentrate, the acid volume must be established by in-process titration to the target.

Preparation follows a 17-step aseptic standard operating procedure: donning of personal protective equipment; cabinet and environment verification; component identity and lot verification; aseptic withdrawal of 5-FU via CSTD; transfer to an amber glass vial; sequential addition of vehicle then acid with mixing; pH measurement and pass/fail decision; quality-control testing; labelling with the provisional 28-day expiry, the in-use instruction and a ‘Do not refrigerate’ auxiliary label; light-protected, childproof packaging; and cytotoxic decontamination and waste disposal.

## 3. Discussion

### 3.1. Clinical Significance and Unmet Need

The absence of a licensed ophthalmic 5-FU product in Hungary, and in most countries, leaves a gap between clinical evidence [1,2,3] and access. This case study sets out the documentation an institution would need in order to reproduce a CM/Res(2016)1-compliant magistral preparation: a structured risk assessment, a quality target product profile, a defined composition and release specification, a standard operating procedure and real-time stability data.

### 3.2. Regulatory Compliance

Compliance with CM/Res(2016)1 is documented by the qualitative risk assessment (Table 1; an alternative method permitted under the Resolution rather than its Note 1 model procedure, Section 4.1), the quality target product profile (Table 2), aseptic compounding from pharmaceutical-grade materials, the evaluation of delivered doses against the criteria of Ph. Eur. 2.9.27 and the real-time storage data [13,14,15,26]; the requirements of Ph. Eur. monograph 1163 for preservative-free multidose presentations are considered in Section 3.5 and Section 3.6 [29].

### 3.3. pH as a Critical Quality Attribute

Commercial 5-FU concentrates are alkaline (pH 8.5–9.5) in order to maintain the drug as its soluble sodium salt, whereas tear-film pH is 7.3–7.6 and is a determinant of ocular comfort [21,23]. Acid titration identified the vehicle-to-acid ratio as the principal critical process parameter (Section 2.1, Table 2). The more fundamental parameter, however, is the acid–base speciation that this ratio controls. As set out in Section 2.3, titration towards physiological pH converts the drug from its freely soluble anionic form to the substantially less soluble un-ionised acid, reducing the solubility margin of a 10 mg/mL preparation to roughly one-third to one-half of the saturation concentration at 20 °C, depending on the literature pKa (Section 2.3), and further on cooling. pH is therefore a critical quality attribute in two distinct senses—ocular tolerability and physical stability—and in this formulation the two pull in opposite directions.

This tension has not, to our knowledge, previously been articulated for compounded 5-FU eye drops. The clinical evidence base for topical 5-FU in OSSN, including the studies cited here [1,2,3], was generated with unbuffered alkaline dilutions of the injectable concentrate, and Antonietti et al. have shown such a preparation to remain stable for 12 months at pH 8.55–8.82, including six months under refrigeration and without precipitation [18]. Alkaline instillates would be expected to be less comfortable, and the burning and irritation commonly reported with topical 5-FU [1,3,4,7] may be attributable in part to pH, although this has not been demonstrated. Adjustment towards the physiological range is thus a rational tolerability objective, but it is not without cost: it narrows the margin against precipitation and removes refrigerated storage as an option.

The present data cannot establish which trade-off is preferable, because ocular tolerability was not measured, nor was osmolality, which also determines comfort (Section 3.9); no claim of superior tolerability is therefore made for the pH-adjusted preparation. Because improved tolerability is the rationale for pH adjustment, this rationale remains a hypothesis until osmolality has been measured and tolerability compared clinically. A comparative tolerability study of pH-adjusted against unadjusted 1% 5-FU eye drops would settle the question, and is the clinical study most directly indicated by this work. Until such data exist, both preparations should be regarded as defensible, and the choice made institutionally on the basis of storage capability and observed patient tolerance.

### 3.4. Delivered Dose, Drop Volume and Clinical Dosing

The mean delivered dose of 0.435 mg per drop corresponds to approximately 1.74 mg/day at four instillations daily, approximately 7 mg per 4-day cycle, most of which is drained or spilled (below). The reproducibility of the delivered dose (RSD 5.1%; all individual doses within ±8% of the mean) supports predictable dosing.

The drop volume itself warrants comment. At 43.5 µL, each instilled drop is four to six times the 7–10 µL capacity of the conjunctival sac, so that most of the dose is cleared through the nasolacrimal duct or spills onto the periocular skin. For a cytotoxic agent this bears on two of the adverse effects reported in the literature: periorbital erythema and oedema, and the punctal and canalicular stenosis described by Alvarez et al. [7]. The punctal occlusion and periocular barrier cream specified in our handling protocol mitigate but do not eliminate this exposure. A dropper delivering 25–30 µL would reduce the drained fraction without reducing the quantity reaching the ocular surface, and merits evaluation; the choice of dropper is therefore not only a dose-uniformity question but a safety one.

### 3.5. Storage Stability and In-Use Instruction

The real-time data are consistent with a 28-day storage period for stored samples at 15–25 °C: assay at or above 97% of the initial value, pH within specification throughout, no visible change, and sterility maintained across the period. No extrapolation beyond the 28 days tested is made. Because these data derive from a single batch with a single determination per timepoint, and from an assay that was not validated as stability-indicating (Section 4.9), the 28-day period is assigned as a provisional storage period for the institutional preparation and is not presented as a formally validated shelf-life; confirmation with replicate determinations on several batches would be required before it could be regarded as such (Section 3.10).

The distinction between storage stability and the permissible in-use period should be maintained. Ph. Eur. monograph 1163 limits the period after first opening a multidose eye-drop container to four weeks unless otherwise justified and authorised [29]; in the absence of in-use microbiological data (Section 3.6), a 28-day in-use period for a preservative-free multidose container is not claimed. No in-use period after opening is derived from the present data, which describe stored samples only. For clinical supply, a precautionary institutional rule is applied instead: because treatment is given in 4-day cycles separated by 3-week intervals, each cycle requires only about 16 drops (about 0.7 mL) per treated eye, so a fresh container is opened at the start of each cycle and discarded at its end, and in any case within 7 days of opening, as in the Moorfields protocol [4]. This rule limits the period of exposure but is not itself supported by in-use microbiological data; it is reported as a handling instruction, not as a demonstrated in-use shelf-life.

### 3.6. Microbiological Assurance

Given the vulnerability of the cornea to infection, and particularly of an ocular surface already compromised by neoplasia and by cytotoxic therapy, sterility is non-negotiable. The combination of aseptic ISO Class 5 compounding, sterility testing according to Ph. Eur. 2.6.1 across the full 28-day period under both storage conditions, and endotoxin testing provided layered assurance against both viable and non-viable microbial hazards, and the preparation remained sterile throughout.

These data characterise the product as stored rather than as handled by the patient. The preparation is preservative-free and is supplied in a conventional multidose dropper that the patient opens four times daily, and Ph. Eur. 1163 requires an antimicrobial preservative in aqueous multidose eye preparations unless the preparation itself has adequate antimicrobial properties within the meaning of Ph. Eur. 5.1.3 [29,30]. The risk this addresses is not theoretical: Roquefeuil et al. recovered contamination from 33% of eye drops used during hospitalisation or returned by patients, with Gram-positive bacteria on 90% of dropper tips and caps [31]. A simulated in-use study and a Ph. Eur. 5.1.3 preservative-efficacy challenge were not performed, and the preparation is not claimed to meet the multidose requirement of Ph. Eur. 1163 through intrinsic antimicrobial properties; an unpreserved aqueous solution would not be expected to pass a challenge test unless the active substance itself were sufficiently antimicrobial, which has not been shown for 5-FU at this concentration. The preservative-free presentation is instead a deliberate, risk-based deviation for a pharmacy preparation, justified by the ocular-surface toxicity of preservatives on an epithelium already compromised by neoplasia and cytotoxic therapy. It is mitigated by the precautionary rule of one container per 4-day treatment cycle, discarded within 7 days of opening (Section 3.5), by patient instruction in dropper hygiene and by supply from the hospital pharmacy under specialist supervision. These measures reduce, but have not been shown to control, the in-use microbiological risk. Unit-dose containers were judged economically impractical at current volumes (Section 2.1), but a multidose container incorporating a microbial filter tip would preserve the economics of the multidose presentation while removing the tip-contamination pathway.

### 3.7. Comparison with the Published Literature

Table 7 places the present preparation alongside those compounded 5-fluorouracil formulations for which pharmaceutical data have been published.

The concentration (1%) and the CM/Res(2016)1-based quality framework align with published clinical and pharmacopeial standards [1,2,3,13,21,22,23]. The target pH of 7.30–7.60 distinguishes this preparation from all published compounded 5-FU eye drops, which are unadjusted and alkaline, as does the use of an ophthalmic vehicle other than saline; the viscosity of the finished preparation was not measured, and the preparation is not described as viscous. The shorter period covered by the present stability data, relative to Antonietti et al. [18], reflects the 28-day duration of the study rather than any observed loss of quality at room temperature; the incompatibility with refrigerated storage, by contrast, is attributed to the pH adjustment rather than to any deficiency in the compounding process, and is plausibly explained by the speciation argument of Section 2.3.

### 3.8. Clinical Implementation Considerations

Safe use depends on the handling measures specified in Section 4.12—supervised instillation training, informed consent, contraception counselling, punctal occlusion, storage and discard instructions, cytotoxic waste handling and ophthalmological follow-up [1,4,7,8].

### 3.9. Strengths and Limitations

Strengths include the systematic application of CM/Res(2016)1 with a documented and reproducible qualitative risk assessment (an alternative method permitted under the Resolution) and a quality target product profile; the comparison of injectable concentrates from five manufacturers, which shows that every concentrate examined requires substantial pH correction; preparation and testing of three consecutive batches; real-time stability data under two storage conditions with an explicit, quantified mechanistic hypothesis for the refrigerated behaviour; and layered microbiological assurance, with sterility maintained across the full 28-day period under both storage conditions.

Limitations include single-institution experience requiring external validation; stability characterised only to 28 days in a single batch with single determinations, with no extrapolation attempted, so that the 28-day storage period is provisional rather than a formally validated shelf-life; the small anticipated patient population, given the rarity of the disease, which precludes conventional process validation on clinical batches; and dependence on a specific three-piece dropper whose delivered volume of 43.5 µL considerably exceeds the conjunctival sac capacity and whose performance may not generalise to other droppers; each concentrate was represented by a single lot and a single pH reading, precluding statistical comparison between manufacturers.

Five further limitations follow from the scope of the analytical and microbiological programme and should be stated explicitly; in each case, the claims of the manuscript have been limited to what the existing data support. First, the HPLC assay was used for comparative trending and was not validated under ICH Q2(R2), nor were forced-degradation studies performed to confirm that it resolves 5-FU from its degradation products [32]; assay results are consequently reported as a percentage of the initial value rather than against the label claim. Second, the precipitate formed at 2–8 °C was neither quantified nor identified and redissolution on re-warming was not tested, so the decline in the refrigerated arm cannot be attributed to chemical degradation as distinct from loss of drug to the precipitate; the handling protocol therefore requires discard of any affected container, whichever mechanism applies. Third, osmolality was not measured; a calculated estimate is given (Section 2.3), but no claim of isotonicity is made. Fourth, viscosity was not measured, and since the vehicle constitutes only 4 mL of the 10 mL batch, no claim is made that the finished preparation prolongs corneal contact time relative to an aqueous solution. Fifth, microbiological testing characterised the preparation as stored; a simulated in-use study and a Ph. Eur. 5.1.3 preservative-efficacy challenge were not performed, so that no in-use period after opening is claimed (Section 3.5). A comparative clinical evaluation against surgery, radiotherapy or unadjusted alkaline 5-FU drops was outside the scope of this study, and only two vehicle-to-acid ratios were examined, without exploration of alternative buffer systems. Further, the characterisation studies were performed on one batch, with single determinations at each stability timepoint, so that batch-to-batch variability is supported only by release compliance (Table S1) and the stability data could be evaluated only descriptively (Section 4.8); precipitated samples were not re-warmed and re-assayed and no unadjusted-pH control was included; Formulation F was not carried into stability testing; and the titration behaviour of concentrates other than Accord near the target pH was not established.

### 3.10. Future Directions

The limitations above define the next steps. Before refrigerated storage or a longer in-use period could be considered, or before the formula is adopted by other institutions, the analytical programme should be extended by validation of the assay under ICH Q2(R2) with forced degradation, by re-warming and re-assay of refrigerated samples with analysis of the precipitate, by an unadjusted-pH (≈8–9) control in the same vehicle and container, and by determination of the solubility of 5-FU in the finished vehicle as a function of temperature; osmolality and viscosity should be measured on the finished preparation, and a simulated in-use microbiological study performed. Beyond this, priorities include multi-institution validation and protocol harmonisation; formal stability studies over 6–12 months with replicate determinations on at least three batches; a comparative ocular tolerability study of pH-adjusted against unadjusted 1% 5-FU eye drops; evaluation of alternative buffer systems, of a lower-volume dropper and of multidose containers with microbial filter tips; prospective clinical outcome registries; and pharmacokinetic studies of ocular and systemic 5-FU exposure.

## 4. Materials and Methods

### 4.1. Risk Assessment Framework

A qualitative, structured risk assessment was conducted in accordance with Council of Europe Committee of Ministers Resolution CM/Res(2016)1 on quality and safety assurance for pharmacy-prepared medicinal products for special patient needs [13,14,15]. The Resolution provides a model procedure for risk assessment in its Note 1, based on the multiplication of numerical risk factors, but permits alternative risk assessment methods, provided that an appropriate assessment of the risk is obtained [13]. The assessment reported here is such an alternative, qualitative method; the Note 1 model procedure was not applied, and no numerical risk score was calculated. Risk parameters evaluated included: route and environment of application (direct ophthalmic instillation, zero contamination tolerance); hazard profile of the active substance (5-FU, ATC L01BC02 [33]); preparation complexity (multi-step aseptic, closed-system process); limited batch volume (≤20 units per year); trained clinical oversight; container and closure integrity; professional conditions of use; formulation stability requiring pH control; pharmaceutical-grade starting materials; and available quality-assurance infrastructure (laminar-airflow cabinets, closed-system transfer devices [CSTDs], standard operating procedures, qualified personnel).

The hazard classification of 5-FU requires precise statement. The International Agency for Research on Cancer places 5-fluorouracil in Group 3, that is, not classifiable as to its carcinogenicity to humans [19]. Its handling as a hazardous medicinal substance is justified not by carcinogenicity but by its cytostatic mechanism, its established teratogenicity and its mutagenic potential, which together place it within the category of medicinal substances requiring closed-system handling. Throughout this manuscript, the substance is accordingly described as hazardous, teratogenic and mutagenic, and not as a carcinogen.

The process was classified as high-risk owing to the ophthalmic route, the hazard profile of the active substance and aseptic complexity, mitigated by controlled-environment compounding, CSTD use, standardised procedures and personnel training. The individual risk parameters and the direction of their contribution to the overall classification are set out in Table 1. The outcome is expressed in the two categories of CM/Res(2016)1 (high-risk and low-risk preparations) [13,15], but it was reached qualitatively from the parameters listed in Table 1 rather than by the Note 1 model procedure. Because the qualitative assessment also considered parameters that are not represented among the factors of the model procedure—the closed-system handling required by the hazard profile of the active substance, the pH-dependent physical stability of the formulation and inappropriate storage by the patient—its outcome need not coincide with a score obtained by that procedure. In this assessment, the combination of the decisive parameters—a sterile ophthalmic preparation, aseptic processing and a hazardous active substance—placed the preparation in the high-risk category, irrespective of the weight given to the mitigating factors; this is also the more conservative classification, since it entails more stringent quality-system requirements [13]. A quality target product profile was defined following the elements of ICH Q8(R2) proportionate to a magistral preparation [20], and the criticality of each quality attribute was assessed (Section 2.1, Table 2).

### 4.2. Source Materials

Active substance: injectable 5-FU concentrates (50 mg/mL) from five manufacturers were obtained and examined for pH: Medac^®^ (medac GmbH, Wedel, Germany; LOT N230721AA), Ebewe/Sandoz^®^ (Sandoz Hungária Kft., Budapest, Hungary; LOT 104754), 5-FROTU^®^ (Onko İlaç San. ve Tic. A.Ş., Kocaeli, Türkiye; LOT 50207610), Teva^®^ (Teva Gyógyszergyár Zrt., Debrecen, Hungary; LOT 10000415) and Accord^®^ (Accord Healthcare Polska Sp. z o.o., Warsaw, Poland; LOT P2500573), all pharmaceutical grade with documented microbiological purity. The first four were compared head-to-head under a fixed acid aliquot (Section 4.5, Table 3); the Accord concentrate was carried into the optimisation series (Table 4).

Excipients: Solvens viscosa pro oculoguttis sine conservante (preservative-free ophthalmic vehicle; institutional formulation, LOT 4248; composition per 100 g: hydroxyethylcellulose 0.40 g, disodium phosphate dodecahydrate 1.50 g, sodium dihydrogen phosphate dihydrate 0.20 g and water for injections to 100.0 g, i.e., the Solvens viscosa pro oculoguttis vehicle of the Hungarian national formulary (Formulae Normales VIII) prepared without its cetrimide preservative [34]) and sterile Infusio Acidi chloridi 0.1 N (0.1 N hydrochloric acid infusion, LOT 250303), both pharmaceutical grade, supplied sterile, with full batch traceability.

Container and closure: the preparation was filled into amber glass bottles closed with a three-piece dropper assembly (bottle, dropper insert and childproof screw cap); the same container type was used for dispensing, for drop-volume testing (Section 4.7) and for the storage-stability samples (Section 4.8).

### 4.3. Batches Prepared

Three consecutive 10 mL batches of the optimised formulation were prepared on separate days under identical conditions and were each subjected to the end-product test programme described below. All three met the release criteria; the release results of each batch are given in Table S1 (Supplementary Material). Sample allocation was as follows: all three batches underwent release testing (Section 4.11), whereas the drop-volume, dose-uniformity and 28-day stability studies were performed on one batch, with a single determination per storage condition and timepoint. The values in Table 5 are therefore individual results, not means, and batch-to-batch consistency is supported by release compliance only (Section 3.9). Representative chromatograms are shown in Figure S1 (Supplementary Material).

### 4.4. Compounding Environment and Personnel Protection

Preparation occurred in the hospital pharmacy’s Cytostatic Admixture Preparation Laboratory, in a Class II biological safety cabinet (ISO Class 5, negative pressure, vertical laminar airflow) using CSTD for handling of the hazardous substance. Personnel wore double nitrile gloves, Tyvek^®^ suits (DuPont, Wilmington, DE, USA), FFP3 or powered air-purifying respiratory protection, and shoe, hair and neck covers, per EMA and OSHA hazardous-drug handling standards.

### 4.5. Pilot Formulation Development and pH Optimisation

Every 10 mL pilot batch comprised 2 mL of injectable 5-FU concentrate at 50 mg/mL, providing 100 mg of 5-FU, together with the vehicle and acid volumes given below, so that all formulations summed to a nominal total volume of 10 mL. Initial pilot formulations (Series A–D) combined the 2 mL concentrate with 8 mL preservative-free vehicle (Series 1) or with 5 mL vehicle and 3 mL 0.1 N hydrochloric acid (Series 2). Optimised formulations (Series E, F) used 2 mL of Accord concentrate with 4 mL vehicle and 4 mL acid, or with 3 mL vehicle and 5 mL acid, respectively. The compositions and the pH obtained are reported in Table 3 (Series A–D) and Table 4 (Series E and F).

### 4.6. pH Measurement

pH was measured at 20–25 °C with a calibrated glass-electrode meter (calibration points pH 4.00, 7.00 and 10.00), under gentle stirring, recording stable values (±0.05 units, ≥30 s). A stepwise correction protocol (0.1–0.2 mL acid increments, re-measurement, documentation) was applied if the measured pH lay outside the target range; formulations requiring excessive correction were rejected in favour of an alternative 5-FU source. Following selection of Formulation E, the release specification for pH was set at 7.30–7.60 (Section 2.2).

No formal estimate of pH measurement uncertainty (for example, from replicate readings of a single sample or of reference buffers) was made. The seven pH values recorded for Formulation E at controlled room temperature (Table 5; mean 7.45, SD 0.036 pH units, range 7.41–7.51) describe the temporal variability of pH across the room-temperature stability measurements. Because they were obtained on different days during storage, this variability may include both genuine changes in the formulation and analytical variation, which the present design cannot separate; it is therefore not treated as measurement uncertainty and is not used to establish a release guard band. Release decisions are made directly against the 7.30–7.60 specification.

### 4.7. Drop Volume and Delivered Dose Determination 

Drop volume and delivered dose were determined gravimetrically, with reference to the Hungarian Pharmacopoeia VIII [35], on the batch used for the stability study (Section 4.3), using the validated three-piece dropper and container described in Section 4.2. Two separate series were performed. (i) Drop-volume series: three runs of 15 consecutive drops, i.e., 45 drops in total, dispensed from a single container; the mass of each drop was recorded on an analytical balance (±0.0001 g) and converted to volume using the measured formulation density (1.002 g/mL), and the mean, SD and RSD were calculated from the 45 individual drop volumes (total mass 1.962 g; Section 2.4). (ii) Delivered-dose series: a separate series of 20 drops from the same container, each weighed individually; the delivered dose of each drop was calculated as concentration (10 mg/mL) × drop volume. Acceptance criteria for the delivered doses were applied by analogy with Ph. Eur. 2.9.27 [26] (not more than 2 of 20 individual doses deviating from the mean by more than 10%, none by more than 20%); the relative standard deviation is reported as a descriptive measure of reproducibility, and individual values were assessed for outliers against the mean ± 3 SD.

### 4.8. Stability Evaluation

The optimised 1% formulation (Series E) was stored at 15–25 °C (intended use) and 2–8 °C (comparator) in the amber glass dropper bottles used for dispensing, protected from light, and assessed at 0, 1, 2, 4, 7, 14 and 28 days for clarity, colour, visible particles, pH and 5-FU content by reversed-phase HPLC-UV. Storage conditions are reported as the nominal ranges of the controlled storage areas used (controlled room temperature, 15–25 °C; pharmaceutical refrigerator, 2–8 °C); individual temperature records are not reported. Freezing was not studied. The acceptance criterion was ≥90% of the initial assay at all timepoints. Because the assay was not validated against the label claim (Section 4.9), this criterion describes retention relative to day 0 only.

To describe the direction and approximate magnitude of change, the tabulated assay values were fitted by ordinary least-squares linear regression on storage time, separately for each storage arm. Because each timepoint is represented by a single determination on one batch, there is no independent estimate of analytical (pure) error, and genuine change, analytical variation and lack of fit cannot be separated; the fitted slopes are therefore reported as exploratory, descriptive estimates only. No hypothesis tests or confidence intervals are reported, no statistical evaluation of the ICH Q1E type [27] was performed, and neither the conclusions nor the assigned storage period rest on the regression; the stability assessment is based on the compliance of the individual results with the acceptance criteria at each timepoint. The pH values of the four concentrates, single readings on a single lot per manufacturer, are reported descriptively (mean, SD, range), as no replicate data were available for inferential comparison.

### 4.9. Analytical Method

5-FU was quantified by reversed-phase HPLC with ultraviolet detection (C18 column, 266 nm; mobile phase 0.1 M phosphate buffer pH 4.5: methanol, 85:15 *v*/*v*), calibrated against a European Pharmacopoeia reference standard (EDQM, Strasbourg, France) of ≥98% purity. The method was used for comparative trending of assay values against the day-zero result within each storage arm. It was not subjected to formal validation under ICH Q2(R2) [32] and forced-degradation studies were not performed; the consequences of this for the interpretation of the stability data are set out in Section 2.5 and Section 3.9. Assay results are accordingly interpreted as comparative trend data within each storage arm; they do not establish content against the label claim, and the method is not claimed to be stability-indicating.

### 4.10. Microbiological Testing

Microbiological testing was performed in accordance with the European Pharmacopoeia. Sterility was assessed by membrane filtration (Ph. Eur. 2.6.1 [28]) using fluid thioglycollate and soybean–casein digest media with 14-day incubation, on samples drawn over the course of the 28-day stability study under both storage conditions. Bacterial endotoxin was measured by the LAL gel-clot assay (Ph. Eur. 2.6.14 [28]). The European Pharmacopoeia does not prescribe an endotoxin limit for topical ophthalmic preparations; the limit of 0.2 EU/mL applied here was adopted as an internal specification by analogy with intraocular preparations, in view of the compromised epithelial barrier expected in the treated eye. Clarity and freedom from visible particles were assessed visually at each stability timepoint.

### 4.11. Quality Control and Batch Documentation

Each batch underwent in-process control (pH, visual inspection) and end-product testing (pH, clarity, sterility, endotoxin, HPLC assay; dose uniformity only on the batch used for the stability study, Section 4.3), with full documentation of lot numbers, personnel, equipment, process parameters and quality-control sign-off; the release results of the three batches are given in Table S1.

### 4.12. Clinical Use Protocol

The dispensed product specified: storage at 15–25 °C protected from light in a childproof container (refrigeration contraindicated on the basis of the stability data; freezing, which was not studied, excluded as a precaution); a provisional storage period of 28 days from preparation for the unopened container (supported by the single-batch storage-stability data; Section 2.5) and, as a precautionary instruction not derived from in-use data, use of each opened container for a single 4-day treatment cycle, with any remainder discarded at the end of the cycle and in any case within 7 days of opening; an auxiliary ‘Do not refrigerate’ label; inspection before each use, with instruction to discard the container, without attempting to redissolve its contents by warming, if any cloudiness or particles are seen; one drop four times daily in 4-day cycles separated by 3-week intervals, typically four cycles [4,6]; application of white soft paraffin to the periocular skin before instillation; punctal pressure for 2 min after instillation; a spacing of at least 5 min from other eye drops; cytotoxic waste disposal; and contraindications (pregnancy and 3 months thereafter, herpetic keratitis or corneal epithelial defects, recent ocular surgery), with mandatory effective contraception during and for 3 months after therapy given the teratogenicity of 5-FU [4,8].

## 5. Conclusions

This work shows that a specialised ophthalmic chemotherapy preparation for a rare indication can be developed and compounded to rigorous pharmaceutical and regulatory standards in a university hospital pharmacy, closing a gap between clinical evidence and product availability. The resulting 1% preservative-free 5-FU ophthalmic solution achieves a pH of 7.43, within the physiological tear-film range and the reported stability optimum for 5-FU; reproducible delivered doses (0.435 ± 0.022 mg per drop, RSD 5.1%; all individual doses within ±8% of the mean); compliant appearance and pH, and retention of at least 97% of the initial assay, over 28 days of storage at 15–25 °C (one batch), which supports a provisional 28-day storage period rather than a formally validated shelf-life; and sterility maintained throughout the study period with a low endotoxin burden, supported by a documented risk assessment, release specification and standard operating procedure.

The principal pharmaceutical finding is that pH adjustment of 5-FU eye drops is not a neutral operation: converting most of the drug from its soluble sodium salt to the un-ionised acid (an estimated 54–79% at pH 7.43, depending on the literature pKa) narrows the solubility margin of a 10 mg/mL preparation, and refrigerated storage led to precipitation; this mechanism is consistent with the data but was not established experimentally. Institutions adopting this formula must therefore store it at controlled room temperature and should regard the 28-day storage period as provisional until it has been confirmed on further batches; an in-use period after opening was not established, and a precautionary rule of one container per 4-day treatment cycle is applied pending in-use data; and the choice between a pH-adjusted and an unadjusted alkaline preparation should be made deliberately, weighing ocular tolerability against storage flexibility. Multi-institutional validation, comparative tolerability data, completion of analytical method validation, measurement of osmolality and viscosity, in-use microbiological data, extended multi-batch stability studies and clinical outcome registries would consolidate this approach and support eventual regulatory harmonisation of 5-FU ophthalmic formulations across European health systems.

## Figures and Tables

**Table 1:** Qualitative structured risk assessment, performed as an alternative method permitted under Council of Europe Resolution CM/Res(2016)1 rather than by the model procedure of Note 1 of the Resolution; no numerical risk score was calculated.

Risk Parameter	Assessment	Contribution
Route and environment of administration	Direct instillation onto the ocular surface; no tolerance for microbial or particulate contamination	Increases risk
Hazard profile of the active substance	Cytostatic antimetabolite; IARC Group 3, i.e., not classifiable for carcinogenicity [19]; teratogenic and mutagenic, requiring closed-system handling	Increases risk
Complexity of the preparation process	Multi-step aseptic process with closed-system transfer, acid titration and in-process pH control	Increases risk
Formulation stability	pH-dependent solubility and stability; requires controlled titration and a defined storage temperature	Increases risk
Batch size and number of units	10 mL per batch; ≤20 units per year—limited population exposure	Mitigates risk
Supervision and conditions of use	Prescribed and monitored by a tertiary ophthalmology service; instillation technique taught to the patient	Mitigates risk
Quality of starting materials	Licensed injectable concentrate and sterile pharmaceutical-grade excipients with full batch traceability	Mitigates risk
Container and closure	Amber glass with three-piece dropper and childproof closure; preservative-free multidose presentation	Mitigates risk; residual concern (Section 3.6)
Quality-assurance infrastructure	ISO Class 5 Class II biological safety cabinet, CSTD, validated SOPs, qualified personnel, documented release testing	Mitigates risk
Storage by the patient	Refrigeration at home despite labelling would cause precipitation and non-uniform dosing of a cytotoxic agent (Section 2.5)	Increases risk; mitigated by labelling, counselling and a discard-if-cloudy instruction (Section 4.12)
Overall classification	High risk (qualitative judgement, expressed in the high-risk/low-risk categories of CM/Res(2016)1; not derived from the Note 1 model procedure), driven by the ophthalmic route, the hazard profile of the active substance and aseptic complexity	—

**Table 2:** Quality target product profile (QTPP) and criticality of quality attributes. CQA, critical quality attribute.

Quality Attribute	Target	CQA	Justification of Criticality	Characterisation in This Study
5-FU content	10 mg/mL (1% *w*/*v*); ≥90% of initial throughout the assigned storage period	Yes	Determines efficacy and the cytotoxic dose delivered per drop	Fixed volumetric composition; HPLC assay relative to day 0 (method not validated; Section 4.9)
pH	7.30–7.60	Yes	Governs ocular tolerability and, through speciation, the solubility margin of 5-FU (Section 2.3)	In-process and release control; measured at every stability timepoint
Appearance	Clear solution, free of visible particles	Yes	Precipitate causes non-uniform dosing; particles may injure the ocular surface	Visual inspection at every timepoint
Osmolality	Close to tear film (≈290–300 mOsm/kg [21])	Yes	Hypo- or hyperosmolar instillates cause discomfort and may damage the epithelium	Not measured; calculated estimate (Section 2.3)
Viscosity	No numerical target	No	No claim of prolonged precorneal residence is made; the vehicle is diluted 2.5-fold; its effect on dosing is captured by drop volume, which is controlled directly	Not measured
Drop volume and delivered dose	Individual doses within ±10% of the mean (Ph. Eur. 2.9.27 criteria, by analogy)	Yes	Determines the dose of a cytotoxic agent per instillation	Gravimetric testing (Section 2.4)
Sterility	Sterile (Ph. Eur. 2.6.1)	Yes	Ophthalmic route; ocular surface compromised by neoplasia and therapy	Tested over 28 days under both storage conditions
Bacterial endotoxin	≤0.2 EU/mL (internal limit)	Yes	Compromised epithelial barrier (Section 4.10)	Tested at compounding
Microbial quality in use	No contamination during the in-use period	Yes	Preservative-free multidose container	Not tested; in-use period not established (Section 3.5)
Physical and chemical stability	All CQAs met throughout the assigned storage period at the labelled storage temperature	Yes	Defines the assigned storage period and storage conditions	28-day real-time study at 15–25 °C and 2–8 °C; one batch, single determinations; provisional storage period only (Section 2.5)

**Table 3:** Initial pilot pH results across four injectable 5-FU concentrate sources under a fixed acid aliquot.

Formulation	5-FU Source	Initial pH	pH After 3 mL 0.1 N HCl	ΔpH
A	Medac (N230721AA)	9.04	8.03	−1.01
B	Ebewe/Sandoz (104754)	9.18	8.10	−1.08
C	5-FROTU (50207610)	9.03	8.07	−0.96
D	Teva (10000415)	8.95	8.16	−0.79

**Table 4:** Optimised pilot formulations (Accord concentrate).

Formulation	Vehicle (mL)	0.1 N HCl (mL)	Measured pH	Outcome
E	4	4	7.43	Selected
F	3	5	7.19	Rejected

**Table 5:** Physical and chemical stability of Formulation E over 28 days.

Timepoint	Appearance/pH (15–25 °C)	Appearance/pH (2–8 °C)	Assay, % of Initial (15–25 °C)	Assay, % of Initial (2–8 °C)
0 d	Compliant, pH 7.45	Compliant, pH 7.45	100.0	100.0
1 d	Compliant, pH 7.48	Compliant, pH 7.49	98.0	99.0
2 d	Compliant, pH 7.41	Compliant, pH 7.44	100.0	96.0
4 d	Compliant, pH 7.42	Opalescent, pH 7.50	97.0	94.1
7 d	Compliant, pH 7.47	Precipitate, pH 7.51	99.0	93.1
14 d	Compliant, pH 7.51	Precipitate, pH 7.46	98.0	91.1
28 d	Compliant, pH 7.43	Precipitate, pH 7.53	97.0	89.1

**Table 6:** Final composition per 10 mL batch (Formulation E).

Component	Quantity
5-Fluorouracil injectable concentrate, 50 mg/mL (Accord, LOT P2500573)	2 mL, providing 100 mg 5-fluorouracil
Solvens viscosa pro oculoguttis sine conservante	4 mL nominal; adjusted to a total volume of 10 mL
Infusio Acidi chloridi 0.1 N	4 mL nominal (Accord concentrate); volume adjusted to reach pH 7.30–7.60
Total volume	10 mL
Final concentration	1% (10 mg/mL)
Final pH	7.43–7.45 (release specification 7.30–7.60)
Storage	15–25 °C, protected from light; do not refrigerate or freeze (freezing not studied; precautionary); discard if cloudy or particles are visible
Storage period and in-use instruction	Provisional storage period (unopened): 28 days at 15–25 °C, based on single-batch stability data; not a formally validated shelf-life. In-use period after opening: not established; precautionary rule of one container per 4-day treatment cycle

**Table 7:** Comparison with published compounded 5-fluorouracil preparations.

Source	Concentration	Vehicle	pH	Storage Evaluated	Stability Reported
Antonietti et al. [18]	1% (10 mg/mL)	Preservative-free 0.9% NaCl	8.55–8.82	2–8 °C 180 d, then 20–24 °C 185 d	12 months, 97.5–103.6% of baseline
Komm et al. [17] (infusion, not ophthalmic)	20–50 mg/mL	0.9% NaCl; 5% dextrose	Not adjusted	2–8 °C; 25 °C/60% RH	15 d in bags; 10–15 d in cassettes, with precipitation
Fuhrman et al. [16]	—	Extemporaneous ophthalmic solution	Not adjusted	—	Reported for a compounded ophthalmic solution
Moorfields protocol [4]	1%	Not stated	Not stated	Not stated	7 d in use
Present work	1% (10 mg/mL)	Preservative-free ophthalmic vehicle (Solvens viscosa) with 0.1 N HCl	7.43	15–25 °C; 2–8 °C	28 d real-time at 15–25 °C (unopened; single batch; provisional); refrigeration contraindicated

## Data Availability

The original contributions presented in this study are included in the article/Supplementary Material. Further inquiries can be directed to the corresponding authors.

## Supplementary Materials

The following supporting information can be downloaded at: https://www.mdpi.com/article/10.3390/ph19101600/s1, Table S1: Release test results of the three consecutive 10 mL batches of Formulation E; Figure S1: Representative HPLC-UV chromatograms of the 1% 5-fluorouracil eye drops (Formulation E).

## Abbreviations

The following abbreviations are used in this manuscript:

5-FU	5-fluorouracil
CM/Res	Committee of Ministers Resolution
CSTD	closed-system transfer device
CPP	critical process parameter
CQA	critical quality attribute
EMA	European Medicines Agency
HPLC	high-performance liquid chromatography
IARC	International Agency for Research on Cancer
ICH	International Council for Harmonisation
LAL	Limulus amebocyte lysate
OSHA	Occupational Safety and Health Administration
OSSN	ocular surface squamous neoplasia
Ph. Eur.	European Pharmacopoeia
QTPP	quality target product profile
RSD	relative standard deviation
SD	standard deviation
SOP	standard operating procedure

## References

[1] Joag M.G., Sise A., Murillo J.C., Sayed-Ahmed I.O., Wong J.R., Mercado C., Galor A., Karp C.L. (2016). Topical 5-Fluorouracil 1% as Primary Treatment for Ocular Surface Squamous Neoplasia. Ophthalmology.

[2] Bakal K., Molugu S., Machakuri K., Bejjanki K.M., Kapoor A.G., Kaliki S. (2024). Efficacy of Topical 5-Fluorouracil in the Management of Ocular Surface Squamous Neoplasia: A Study of 101 Eyes. Int. Ophthalmol..

[3] Kato J.M., Ballalai P.L., de Lima P.P., Santo R.M. (2024). Efficacy and Safety of Topical 0.5% 5-Fluorouracil as Primary Treatment of Ocular Surface Squamous Neoplasia. Can. J. Ophthalmol..

[4] Moorfields Eye Hospital (NHS) Fluorouracil (5-FU) Eye Drops. https://www.moorfields.nhs.uk/for-patients/information-hub/fluorouracil-5-fu-eye-drops.

[5] Höllhumer R., Michelow P., Libhaber E., Williams S. (2025). Management and Outcomes of Ocular Surface Squamous Neoplasia at a Tertiary Hospital, South Africa. Eye.

[6] Al-Barrag A., Al-Shaer M., Al-Matary N., Al-Hamdani M. (2010). 5-Fluorouracil for the Treatment of Intraepithelial Neoplasia and Squamous Cell Carcinoma of the Conjunctiva, and Cornea. Clin. Ophthalmol..

[7] Alvarez O.P., Zein M., Serrano A., Galor A., Karp C.L. (2024). Punctal and Canalicular Stenosis Following Topical 1% 5-Fluorouracil Eye Drop Therapy for Ocular Surface Squamous Neoplasia. Cornea.

[8] Tsatsos M., Delimitrou C., Tsinopoulos I., Ziakas N. (2025). Update in the Diagnosis and Management of Ocular Surface Squamous Neoplasia (OSSN). J. Clin. Med..

[9] National Institute of Pharmacy and Nutrition Summary of Product Characteristics: Fluorouracil-TEVA 50 mg/mL Oldatos Injekció.

[10] National Institute of Pharmacy and Nutrition Summary of Product Characteristics: 5-Fluorouracil Sandoz 50 mg/mL Koncentrátum Oldatos Infúzióhoz.

[11] National Institute of Pharmacy and Nutrition Summary of Product Characteristics: Verrumal Külsőleges Oldat.

[12] National Institute of Pharmacy and Nutrition Summary of Product Characteristics: Efudix 50 mg/g Kenőcs.

[13] Committee of Ministers, Council of Europe (2016). CM/Res(2016)1. Resolution on Quality and Safety Assurance Requirements for Medicinal Products Prepared in Pharmacies for the Special Needs of Patients (Succeeding Resolution CM/ResAP(2011)1).

[14] CM/Res(2016)1—Az Európa Tanács Miniszteri Bizottságának Határozata a Betegek Speciális Szükségleteinek Kielégítése Céljából Gyógyszertárban Készített (Magisztrális) Gyógyszerek Minősége és Ártalmatlansága Biztosításának Feltételeiről. https://ogyei.gov.hu.

[15] Scheepers H.P.A., Neerup Handlos V., Walser S., Schutjens M.D.B., Neef C. (2017). Impact of the Council of Europe Resolution on Quality and Safety Assurance Requirements for Medicinal Products Prepared in Pharmacies for the Special Needs of Patients. Eur. J. Hosp. Pharm..

[16] Fuhrman L.C., Godwin D.A., Davis R.A. (2000). Stability of 5-Fluorouracil in an Extemporaneously Compounded Ophthalmic Solution. Int. J. Pharm. Compd..

[17] Komm C., Rochani A., Fox T., Kaushal G. (2022). Stability of Extemporaneously Compounded 5-Fluorouracil Utilizing High Performance Liquid Chromatography. Drug Discov. Ther..

[18] Antonietti M., Theotoka D., Djulbegovic M.B., Gorman G.S., Massey M., Galor A., Karp C.L. (2026). Prolonged Stability of 1% 5-FU Eye Drops as Topical Chemotherapy for Ocular Surface Squamous Neoplasia. Ocul. Surf..

[19] International Agency for Research on Cancer (1987). 5-Fluorouracil (Group 3). IARC Monographs on the Evaluation of Carcinogenic Risks to Humans, Overall Evaluations of Carcinogenicity, Supplement 7.

[20] International Council for Harmonisation (2009). ICH Harmonised Tripartite Guideline Q8(R2): Pharmaceutical Development.

[21] Iyamu E., Enobakhare O. (2019). pH and Osmolality of Pre-Corneal Tear Film and Commercially Available Artificial Tears. EC Ophthalmol..

[22] Ibrahim R.A.A.-Z., Suhail F.S.A., Al-Hakeim H.K. (2018). Stability of Anticancer Drug 5-Fluorouracil in Aqueous Solution: An Assessment of Kinetic Behavior. Nano Biomed. Eng..

[23] Chang A.Y., Purt B. (2025). Biochemistry, Tear Film. StatPearls.

[24] National Center for Biotechnology Information PubChem Compound Summary for CID 3385, Fluorouracil. https://pubchem.ncbi.nlm.nih.gov/compound/Fluorouracil.

[25] Mioduszewska K., Dołżonek J., Wyrzykowski D., Kubik Ł., Wiczling P., Sikorska C., Toński M., Kaczyński Z., Stepnowski P., Białk-Bielińska A. (2017). Overview of Experimental and Computational Methods for the Determination of the pKa Values of 5-Fluorouracil, Cyclophosphamide, Ifosfamide, Imatinib and Methotrexate. TrAC Trends Anal. Chem..

[26] EDQM, Council of Europe (2022). European Pharmacopoeia, General Chapter 2.9.27: Uniformity of Mass of Delivered Doses from Multidose Containers.

[27] International Council for Harmonisation (2003). ICH Harmonised Tripartite Guideline Q1E: Evaluation for Stability Data.

[28] EDQM, Council of Europe (2022). European Pharmacopoeia, General Chapters 2.6.1 (Sterility) and 2.6.14 (Bacterial Endotoxins).

[29] EDQM, Council of Europe (2022). European Pharmacopoeia, Monograph 1163: Eye Preparations.

[30] EDQM, Council of Europe (2022). European Pharmacopoeia, General Chapter 5.1.3: Efficacy of Antimicrobial Preservation.

[31] Roquefeuil L., Iskandar K., Roques C., Marchin L., Guittard M., Poupet H., Brandely-Piat M.-L., Jobard M. (2024). Evaluating and Managing the Microbial Contamination of Eye Drops: A Two-Phase Hospital-Based Study. Pharmaceutics.

[32] International Council for Harmonisation (2023). ICH Harmonised Guideline Q2(R2): Validation of Analytical Procedures.

[33] ATC/DDD Index; WHO Collaborating Centre for Drug Statistics Methodology. https://atcddd.fhi.no/atc_ddd_index/?code=L01.

[34] National Center for Public Health and Pharmacy (NNGYK) (2021). Formulae Normales Editio VIII (FoNo VIII): Solvens Viscosa pro Oculoguttis.

[35] National Institute of Pharmacy (OGYI) (2004). Magyar Gyógyszerkönyv VIII (Hungarian Pharmacopoeia, 8th ed.).

