# Supplementary material for: Formulation Development of a pH-Adjusted, Preservative-Free 1% 5-Fluorouracil Eye Drop Compounded from Injectable Concentrate: A Risk-Assessed Magistral Formulation and 28-Day Stability Case Study
Source: Pharmaceuticals (Basel). 2026 Oct 9;19(10):1600. doi: 10.3390/ph19101600 (PMC13708713; doi:10.3390/ph19101600)
Supplement: Supplementary file 1 [file pharmaceuticals-19-01600-s001.zip › pharmaceuticals-4590262-supplementary.pdf]

Supplementary Materials

Formulation Development of a pH-Adjusted, Preservative-Free 1% 5-Fluorouracil Eye Drop Compounded from Injectable Concentrate: A Risk-Assessed Magistral Formulation and 28-Day Stability Case Study

Péter Vajda[1], Gábor Bartus[1], Máté Csapó[1], István Horváth[1], Márton Vajna[1], Szilvia Sebők[1*], Romána Zelkó[1*]

[1] University Pharmacy Department of Pharmacy Administration, Faculty of Pharmacy, Semmelweis University, Hungary, 1092 Budapest, Hőgyes Endre u. 7-9., vajda.peter@semmelweis.hu

* Correspondence: szilvia.sebok@semmelweis.hu; romana.zelko@semmelweis.hu

Table S1. Release test results of the three consecutive 10 mL batches of Formulation E.

Test (method)	Acceptance criterion	Batch 1[a]	Batch 2[b]	Batch 3[b]
Appearance (visual inspection)	Clear solution, free of visible particles	Clear, colourless to slightly yellow; no visible particles	Complies	Complies
pH (glass electrode, 20–25 °C)	7.30–7.60	7.45	Complies	Complies
5-FU content (composition; HPLC-UV)	10 mg/mL (1% w/v), by fixed volumetric composition[c]	Complies; HPLC-UV day-0 reference (100.0%)	Complies	Complies
Uniformity of delivered dose (gravimetric, 20 drops)	Ph. Eur. 2.9.27 criteria, by analogy: ≤2 of 20 doses deviate >10% from the mean, none >20%	Complies: 0.435 ± 0.022 mg per drop (range 0.40–0.47 mg; RSD 5.1%)	Not tested[d]	Not tested[d]
Sterility (membrane filtration, Ph. Eur. 2.6.1)	Sterile	Sterile	Complies	Complies
Bacterial endotoxins (LAL gel-clot, Ph. Eur. 2.6.14)	≤0.2 EU/mL (internal limit)	<0.05 EU/mL	Complies	Complies

[a] Batch used for the drop-volume, dose-uniformity and 28-day stability studies (Section 4.3); pH and appearance are the day-0 values of the stability study (Table 5), dose uniformity is reported in Section 2.4, and sterility and bacterial endotoxins in Section 2.6. [b] Complies: the batch met the acceptance criterion; all three batches met the release criteria (Section 4.3). [c] Content is defined by the fixed volumetric composition (Table 6); the HPLC-UV assay was used for comparative trending relative to day 0 and was not validated against label claim (Section 4.9). [d] Dose uniformity was determined on one batch only (Section 4.3).

Section and table numbers refer to the article. 5-FU, 5-fluorouracil; EU, endotoxin units; HPLC-UV, high-performance liquid chromatography with ultraviolet detection; LAL, Limulus amebocyte lysate; Ph. Eur., European Pharmacopoeia; RSD, relative standard deviation.

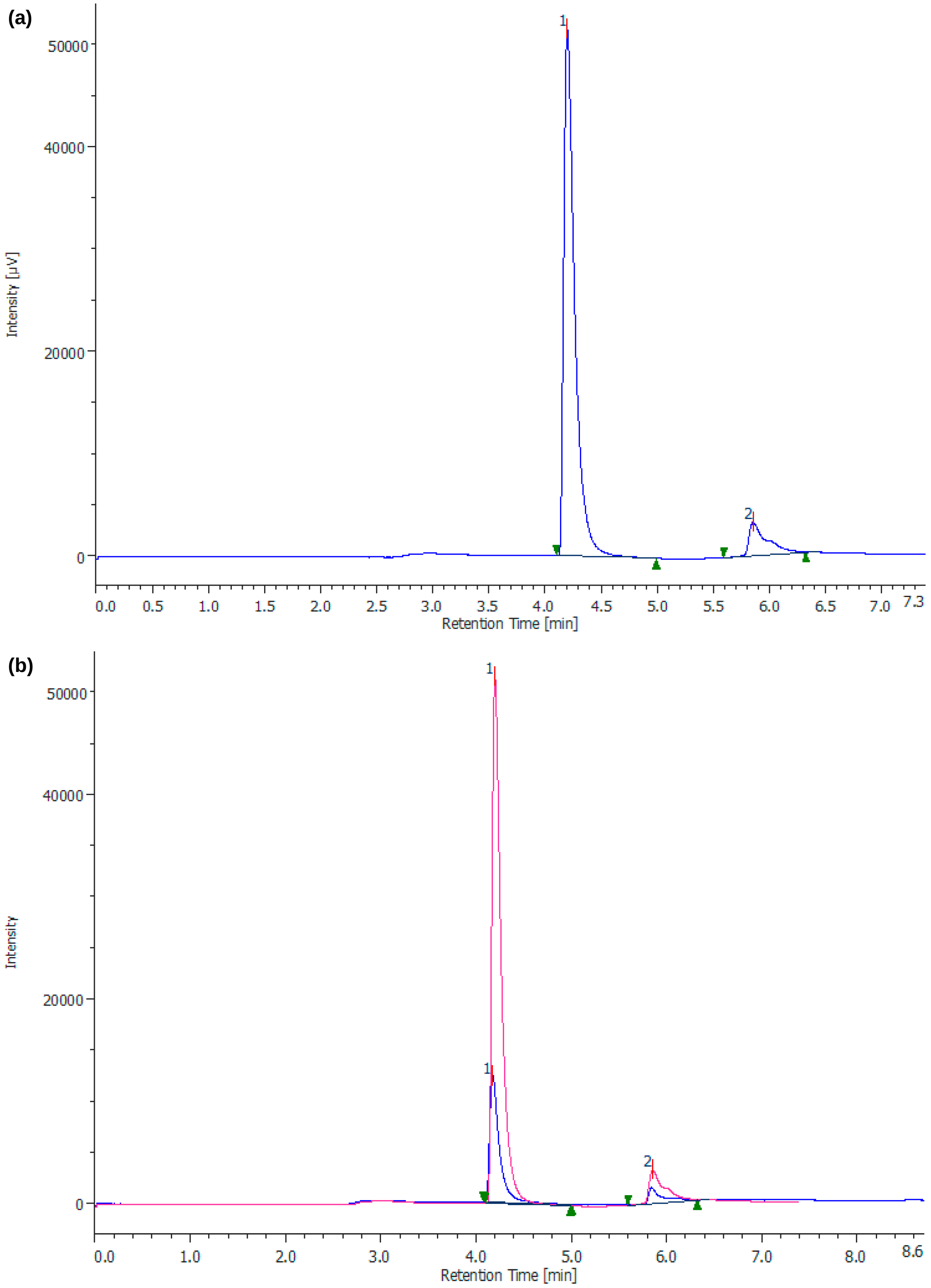

Figure S1. Representative HPLC-UV chromatograms of the 1% 5-fluorouracil eye drops (Formulation E). (**a**) Chromatogram of a Formulation E sample with peak integration; (**b**) overlay of the same chromatogram (magenta) with the chromatogram of the 5-fluorouracil reference standard solution (European Pharmacopoeia reference standard, ≥98% purity; blue). Peak 1, 5-fluorouracil (retention time, $t_R \approx 4.2$ min); peak 2, unidentified ($t_R \approx 5.8$ min). Green triangles mark the start and end of peak integration, and red ticks mark the peak apices. Conditions: C18 column; mobile phase 0.1 M phosphate buffer pH 4.5 : methanol (85:15, v/v); UV detection at 266 nm. Intensity is the detector response in µV in both panels.
